# The Influence of Low Shear Microbore Extrusion on the Properties of High Molecular Weight Poly(l-Lactic Acid) for Medical Tubing Applications

**DOI:** 10.3390/polym11040710

**Published:** 2019-04-18

**Authors:** Brian Dillon, Patrick Doran, Evert Fuenmayor, Andrew V. Healy, Noel M. Gately, Ian Major, John G. Lyons

**Affiliations:** 1 Materials Research Institute, Athlone Institute of Technology, Dublin Road, Bunnavally, Athlone and Co., Westmeath, Ireland; A00237890@student.ait.ie (B.D.); patrickdoran@ait.ie (P.D.); e.fuenmayor@research.ait.ie (E.F.); andrewhealy@research.ait.ie (A.V.H.); n.gately@ait.ie (N.M.G.); imajor@ait.ie (I.M.); 2 Faculty of Engineering and Informatics, Athlone Institute of Technology, Dublin Road, Bunnavally, Athlone, Co., Westmeath, Ireland

**Keywords:** Bioabsorbable polymers, poly-l-lactic acid, microbore extrusion, low shear, residence time, molecular weight, residual monomer, crystallinity

## Abstract

Biodegradable polymers play a crucial role in the medical device field, with a broad range of applications such as suturing, drug delivery, tissue engineering, scaffolding, orthopaedics, and fixation devices. Poly-l-lactic acid (PLLA) is one of the most commonly used and investigated biodegradable polymers. The objective of this study was to determine the influence low shear microbore extrusion exerts on the properties of high molecular weight PLLA for medical tubing applications. Results showed that even at low shear rates there was a considerable reduction in molecular weight (M_n_ = 7–18%) during processing, with a further loss (M_n_ 11%) associated with resin drying. An increase in melt residence time from ~4 mins to ~6 mins, translated into a 12% greater reduction in molecular weight. The degradation mechanism was determined to be thermal and resulted in a ~22-fold increase in residual monomer. The differences in molecular weight between both batches had no effect on the materials thermal or morphological properties. However, it did affect its mechanical properties, with a significant impact on tensile strength and modulus. Interestingly there was no effect on the elongational proprieties of the tubing. There was also an observed temperature-dependence of mechanical properties below the glass transition temperature.

## 1. Introduction

Bioabsorbable polymers have a broad range of applications in the medical device field; this is due to their biocompatibility, biodegradability, and bioresorbability. Applications include but are not limited to suturing, drug delivery, tissue engineering, scaffolding, orthopaedics, and fixation devices. A significant proportion of the biodegradable polymers used and studied today, come from the family of aliphatic polyesters, such as poly(lactic acid) (PLA), poly(glycolic acid) (PGA), poly(ε-caprolactone) (PCL) and their copolymers. This is due to the presence of an ester covalent bond with a reactive polar nature, which can be easily broken down through hydrolysis [1]. Poly (lactic acid) (PLA) is a semi-crystalline aliphatic polyester and is derived from renewable resources such as sugarcane or cornstarch. The synthesis of PLA can be performed through either direct polycondensation or the more preferred method of ring-opening polymerisation (ROP). PLA has ~37% crystallinity, with a glass transition temperature (T_g_) of 60–65 °C and melting temperature (T_m_) of 175–180 °C. PLA is a chiral molecule, which can be found in two stereoisomer forms l and d, giving poly-l-lactic acid (PLLA) and poly-d-lactic acid (PDLA) [2,3].

**Figure 1:**
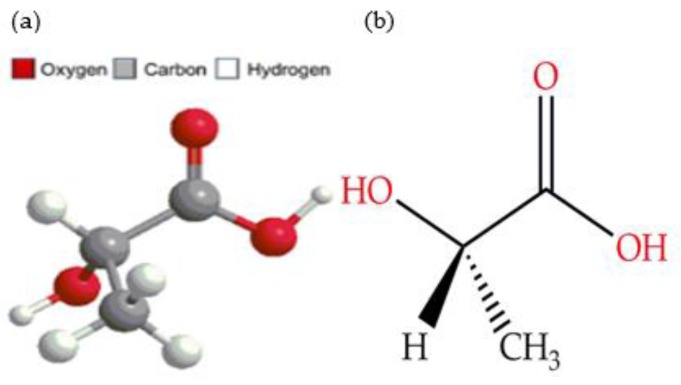
(**a**) Molecular structure of lactic acid, (**b**) Chemical structure of l-(+)-lactic acid.

PLLA is the most widely used form of PLA in biomedical applications since it is the naturally occurring form of lactic acid found in the body. It is described as being a brittle material with a strain of <5% and having a moderate tensile strength of 60–70 MPa, with an elastic modulus of 2–4 GPa [4]. The chemical structure for PLLA is represented in Figure 1.

PLLA can be processed using traditional thermoplastic melt processing techniques such as extrusion, injection moulding, solvent spinning, and casting. As with any viscoelastic material, the properties of PLLA are dependent on time, temperature and strain rate. It behaves like a glassy polymer at room temperature, given it is below T_g_ [5] and is prone to physical aging [6]. The thermal properties of PLLA can be affected by molecular weight and composition (stereoisomer content) [7]. Crystallinity also plays an important role in determining the materials thermal and mechanical properties, as well as its degradation rate [8,9]. The crystallites acting as physical crosslinks stabilize the amorphous phase of the polymer [10] and slow the ingress of water during hydrolytic degradation [11]. PLLA’s melt rheological properties have a profound effect on how the polymer flows during the extrusion process and is dependent on factors such as temperature, molecular weight, and shear rate, all of which require careful consideration during melt processing [10]. PLLA is extremely sensitive to molecular weight loss during melt processing, with the degree of weight loss influenced by processing conditions such as residence time, resin moisture content, and melt temperature. Aside from PLLA’s susceptibility to hydrolytic degradation, one of the major challenges during melt processing is the risk of thermal degradation. The reduction in molecular weight leads to a change in the materials mechanical properties [12,13,14]. Depolymerisation of PLLA during extrusion can increase the percentage monomer and low molecular weight ester oligomers in the material. Impurities such as post-processing residual monomer or other additives have been shown to increase the degradation rate of PLLA [15,16]. The initial molecular weight of PLLA resin is critical, particularly for load bearing medical device applications such as stenting and orthopedics. The initial molecular weight of the polymer must be high enough (typically M_w_ = 300,000–600,0000 g/mol) to withstand the molecular weight loss witnessed during melt processing and sterilisation, which can range from 5 to 88% [10].

Whilst the effect of melt processing on the properties of PLLA has been extensively researched, this work has pre-dominantly involved the use of twin-screw extruders and lower molecular weight (M_w_ < 200,000 g/mol) resin. Twin screw extruders typically generate higher shear rates and viscous heat than microbore extruders. Microbore extruders are typically classified as having a barrel diameter of 1/2” to 1”. Also, the vast majority of post-processing mechanical analysis has been performed on compression or injection moulded samples. Thus, not fully assessing the impact that molecular orientation imparted during tube extrusion plays on the materials mechanical properties. One exception to this was a study on the effect of processing of PLLA [14], where a high molecular weight grade of PLLA resin was extruded into 2-mm rods and its properties characterised. Unfortunately, with very little information provided on the actual processing conditions (shear rate, residence time) and mechanical analysis only looking at shear strength, it is difficult to ascertain the applicability for microbore medical tubing applications.

The aim of this study was to investigate the influence low shear microbore single screw extrusion exerts on the properties of a high molecular weight (M_w_ > 400,000 g/mol) GMP grade bioabsorbable PLLA. A series of analytical techniques were used to assess the effect melt processing had on the materials thermal, chemical, morphological and mechanical properties and its suitability for load bearing medical applications.

## 2. Materials and Methods

### 2.1. Materials

The material used was a poly-l-lactic acid (PLLA), which was obtained from Corbian (Purac, Amsterdam, the Netherlands). It was medical grade homopolymer of L-lactide with the chemical formula (C_6_H_8_O_4_). It has a molecular weight of M_n_ 325,000 and M_w_ 650,000, an inherent viscosity specification of 3.8 dl/g, max residual monomer content of 0.1 wt.% and was supplied in the form of white granules.

### 2.2. Processing

**Figure 2:**
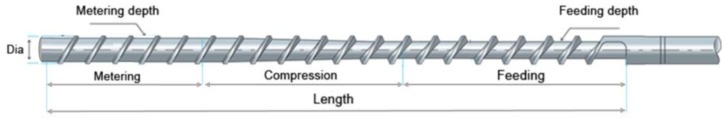
Extrusion Screw Schematic.

For the purpose of this study, two batches of extruded tubing were manufactured. Both batches were processed using different extrusion tooling configurations, to assess if differences in residence time in the extruder (American Kuhne, York, PA, USA) had an impact on the properties of the tubing. The first batch (identified as Batch A) was processed on a 1” extruder barrel with a 24:1 l/d ratio and used a conventional straight flight metering screw design with a 3.1:1 compression ratio. It also incorporated a melt pump, which helped to regulate output, but led to a small increase in residence time of the melt. The second batch (identified as Batch B) was processed on a 0.75” extruder barrel with a 24:1 l/d ratio. It also used a metering screw design with a 3:1 compression ratio, but did not incorporate a melt pump. Figure 2 and Table 1 provide the relevant screw geometry details. There was a 33% difference in melt residence time between extrusion Batch A and B, with 5 min 58 s and 4 min 2 s, respectively. Melt residence time was measured by introducing a pigment tracer into the feed throat and measuring the time it took to exit the die.

**Figure 3:**
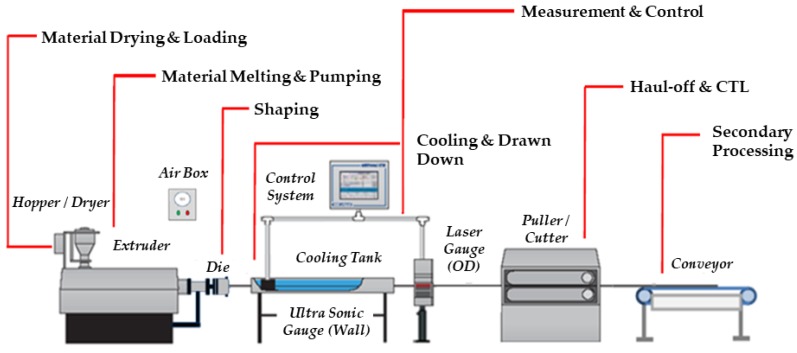
Schematic of tube extrusion process including an extruder and down-stream ancillary equipment.

Given the low shear rates generated in a microbore extrusion process, it is unlikely that differences in the two screw designs or the addition of a melt pump for Batch A would have a significant impact on the rheological properties of the material. Using the formula below, the shear rate of the flight depth in the metering section of both screws was calculated. A shear rate of 28 s^−1^ was calculated for the 1” screw used in Batch A, and a 24 s^−1^ shear rate for the 0.75” screw used in Batch B. Therefore, any differences noted between the material characteristics of Batch A versus B, could most likely be attributed to differences in their melt residence times.

(1) $$\dot{\Upsilon }=\frac{\pi \times D\times N}{60\times h}$$

where $\dot{\Upsilon }$ = Shear rate in screw channel (s^−1^), D = Screw diameter (mm), N = Screw Speed (rev/min) and h = Screw channel depth in metering section (mm) [17]. An extrusion temperature profile of 219 °C in the barrel and increasing to 230 °C in Die was utilised, with a puller speed of 15 ft/min. The use of an infrared camera confirmed there was no difference in melt temperature between both extrusion batches. Screw speed varied from 25 rpm for Batch A to 30 rpm for Batch B. Prior to processing the resin was dried at 50 °C (below the materials T_g_) for 16 hours to a moisture content of <100 ppm. Extruder output was less than 0.5 lbs/hr for both batches. The extruded tubing had an outside diameter of 1.9 mm and 0.6 mm wall thickness. Figure 3 provides a basic schematic of a tube extrusion process.

### 2.3. Rheological Properties

Capillary rheology was performed using a Dynisco LCR7001 (Heilbronn, Germany). The die (Z349-15) used had a 1mm diameter and 15 mm length. The melt flow behaviour in the capillary rheometer was investigated at 219 °C, with shear rate measurements taken between 12 and 3033 s^−1^ on 12, 22, 41, 76, 141, 260, 481, 889, 1642 and 3033 s^−1^. The analysis was performed to evaluate the effect of shear rate on melt viscosity. The PLLA pellets were pre-dried as described in Section 2.2 and then melted in the barrel for 5 min. The output is a viscosity curve plotting apparent shear rate by apparent shear viscosity. No corrections were made to the viscosity-shear rate flow curve, as the analysis was only concerned with the Newtonian flow behaviour in the zero-shear viscosity region of the melt.

### 2.4. Molecular Weight and Chemical Analysis

#### 2.4.1. Molecular Weight

Gel permeation chromatography (GPC) was used to determine the molecular weight of the extruded tubing. The analysis was carried out using a Polymer Laboratories PL GPC-120 system (Shropshire, UK) equipped with an auto-sampler and in-built differential refractometer detector. The samples were prepared by dissolving 50 mg of sample in 5 mL of tetrahydrofuran (THF) to provide a final concentration of 1 mg/mL. The system was fitted with a PL-gel 5 μm Mixed D column (300 × 7.5 mm, Polymer Laboratories). The GPC system was calibrated using a mixture of linear polystyrene standards (M_w_ 162–6,000,000) which were procured from Agilent Technologies (Santa Clara, CA, USA). The molecular weight averages calculated by the system were the number average molecular weight (M_n_), the weight average molecular weight (M_w_) and the polydispersity index (PDI), which is calculated from the simple formulae of M_w_/M_n_.

#### 2.4.2. Chemical Analysis

Fourier-transform infrared spectroscopy (FTIR) was used to provide spectra analysis of the dried PLLA resin and extruded tubing from Batch A. FTIR spectra were recorded using a Thermo Electron Corporation Nicolet 5700 FTIR spectrometer (Madison, WI, USA) equipped with a universal attenuated total reflectance. The spectra were recorded between 4000 and 500 cm^−1^ frequency ranges. The number of scans was 64, with a wavelength resolution of 4 cm^−1^.

#### 2.4.3. Residual Monomer

Gas chromatography-flame ionization detection (GC-FID) was performed on the dried PLLA resin and extruded tubing from Batch A and B, using Agilent 6890 GC with the HP7673A autosampler attached (Wilmington, DE, USA). The system used a DB-17MS, 20 m × 0.18 mm × 0.18 μm column, with split/splitless, injector at 200°C. The injector volume was 1μL, with Helium, 1.3 mL/min, 10:1 split carrier gas. The column temperature program comprised of 50 °C for 1 min, 25 °C/minute ramp to 320 °C and held for 5 min. The detector temperature was set at 335 °C. GC-FID was used to determine the percentage of residual lactide monomer in the samples.

### 2.5. Thermal and Morphological Properties

#### Differential Scanning Calorimetry

Thermal and morphological properties were determined by a DSC instrument (DSC 2920, TA Instruments, New Castle, DE, USA). The samples (8–12 mg) were heated from 30 to 250 °C at a rate of 10 °C/min under a constant nitrogen flow rate of 30 mL/min. The specific heat change (ΔC_p_), glass transition temperature (T_g_), melt recrystallisation temperature (T_mc_), melting temperature (T_m_), cold crystallization temperature (T_cc_), enthalpy of melting (ΔH_m_) and enthalpy of cold crystallisation (ΔH_c_) were determined during the first heating cycle to establish the influence of thermal history during processing. All indicated temperatures are peak temperatures. The degree of crystallinity (X_c_) was calculated using the enthalpy (heat) of melting ΔH_m_ and the enthalpy of cold crystallization ΔH_c_ (where present), through the following equation;

(2) $$X_{c}\;\left(\%\right)\;=\;\frac{\Delta H_{m}\;-\;\Delta H_{c}}{\Delta {H_{m}}^{0}}\;\times \;100$$

Here a ΔH_m_^0^ = 93 J/g is used as the enthalpy of fusion for a 100% crystalline PLLA [18].

### 2.6. Mechanical Properties

#### 2.6.1. Tensile Testing

Uniaxial tensile testing was carried out using a Lloyd LRX tensometer, equipped with a 2.5 kN load cell (Bognor Regis, UK). The gauge length for each specimen was set to 65 mm, with parts subjected to an extension rate of 50 mm/min up to the point of failure. This allowed for the evaluation of Young’s (elastic) modulus, maximum tensile stress, strain at maximum load and strain at break. An average value of 6 samples in each group was presented with their standard deviation. Statistical analysis was performed using a 2-sample T-Test. Tests were conducted using Minitab 17 software. P values of less than 0.05 were deemed statistically significant. The sample size selected was based off an 80% power and capable of detecting a practically significant difference in the means. Practical significance was determined for each attribute by applying engineering knowledge of the process output requirements.

#### 2.6.2. Dynamic Mechanical Analysis

Dynamic mechanical analysis (DMA) was performed using TA Instruments DMA Q800 (New Castle, DE, USA). The test was performed in tension mode using a frequency of 1Hz and an amplitude of 15μm. The temperature range between 0 and 140 °C with a 5 °C/min ramp rate was utilised to determine the storage modulus (E’). A representative DMA plot from 3 samples tested in each group was presented.

## 3. Results and Discussion

### 3.1. Rheological Results

The rheology of PLLA melts has been extensively studied [19,20,21,22,23]. PLLA melts are a non-Newtonian fluid and due to their shear thinning behaviour, see a reduction in viscosity with increasing shear rates. During extrusion, higher shear rates are caused by extruding faster through the die or reducing the barrel wall or die clearance. However, melt viscosity remains constant at low shear rates, known as the zero-shear viscosity region. The zero-shear viscosity of polymers depends on their molecular weight, due to chain entanglement above a critical molecular weight value. For PLLA, the critical molecular weight for entanglement is estimated to be near 9000 g/mol, whilst between entanglements is closer to 4000 g/mol. Similar to many well entangled linear polymers, the relationship of zero shear viscosity is proportional to 3.4–4.0 power of the molecular weight [21,22,23].

**Figure 4:**
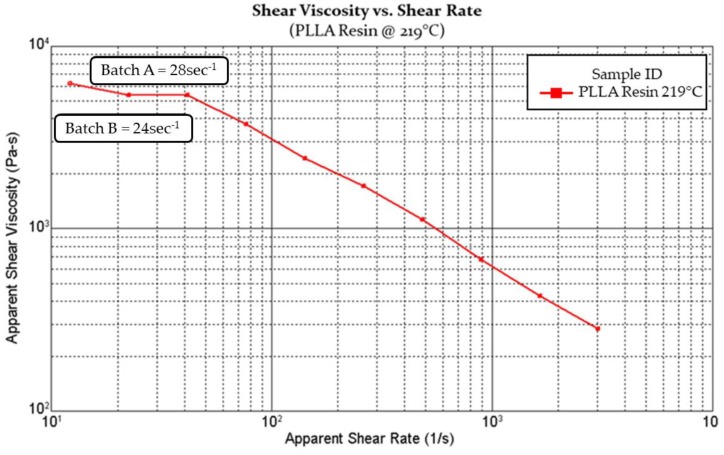
Capillary rheology curve of apparent shear viscosity vs. apparent shear rate.

Capillary rheology was performed on the dried resin prior to processing. Figure 4 provides a capillary rheology curve of the apparent shear viscosity versus the apparent shear rate. The curve highlights that the shear rates calculated in Section 2.1 (Batch A = 28 s^−1^; Batch B = 24 s^−1^) fall within the materials zero shear region and would therefore not effect melt viscosity. Like most thermoplastic polymers, PLLA exhibits Newtonian behaviour at low shear rates (<10 s^–1^) and non-Newtonian behaviour (shear thinning) at the high shear rates (>10 s^–1^), as well a reduction in viscosity with increasing processing temperatures [7].

The heat required to plasticise a polymer during extrusion comes from a combination of conductive heat from the barrel wall and viscous dissipation through the shearing action of screw rotation. Whilst viscous dissipation typically accounts for 80–90% of polymer melting in a conventional extruder under normal operating conditions [24], its plasticising effect in microbore extrusion is negligible. Heat conduction dominates plasticisation in small diameter extruders ran at slow screw speeds [25]. Therefore, the barrel and die temperatures, along with the materials molecular weight are critical in controlling the melt viscosity in microbore extrusion.

### 3.2. Molecular Weight and Chemical Analysis

#### 3.2.1. Gel Permeation Chromatography

Gel permeation chromatography (GPC) analysis was carried out on both batches of extruded tubing to determine the effect that different processing conditions had on the molecular weight and molecular weight distribution of the polymer. For comparison, samples from the as received (undried) and dried PLLA resin pellets were also analysed. Table 2 presents the molecular weight values for all samples.

There was a notable reduction in M_n_ and to a lesser degree in M_w_ observed during the resin drying and extrusion processes, as well as a corresponding increase in PDI. The as received (undried) resin saw a reduction in M_n_ of 11.2% and 2.5% in M_w_ during the drying process. This trend continued during the processing of extrusion Batch A, with a further 17.7% reduction in M_n_ and 12.4% in M_w_. However, the same degree of molecular weight loss was not witnessed for Batch B, whereby there was only a 6.9% M_n_ and 1.5% M_w_ reduction from the extruded material compared to the dried material. The increase in PDI from 2.16 in the undried resin up to 2.5 in the extruded resin, can be attributed to random chain scission resulting in an increase in lower molecular weight chains and an overall broader distribution [26].

**Figure 5:**
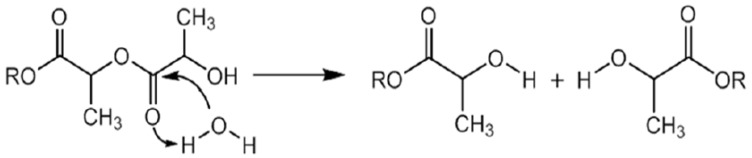
Hydrolytic degradation of PLLA, reproduced from [28] under open access license.

Degradation during melt processing is typically through hydrolytic or thermal degradation mechanisms, resulting in chain scission. The presence of moisture in the raw resin prior to processing leads to hydrolysis of the ester bonds. Figure 5 provides a schematic of the hydrolytic degradation mechanism of PLLA, where hydrolysis produces shorter chains with an increase in the hydroxyl and carboxylic acid end-group [27].

Thermal degradation in PLLA has been attributed to a combination of the following; zipper like depolymerization, oxidative chain scission, intermolecular transesterification to monomer and oligomeric esters and intramolecular transesterification resulting in the formation of monomer and oligomer lactides of low molecular weight [29]. In comparison to other polymers, polylactic acid has a low thermal degradation activation energy (21–23 kJ/mol), indicating it’s sensitivity to thermal processing [30]. Resin moisture content, melt temperature, shear force, melt residence time and residual catalyst in the resin can all impact the level of molecular weight loss experienced during the extrusion of PLLA, with thermal degradation typically seen at temperatures over 200 °C [10].

Given the fact that the processing conditions for both extrusion batches were almost identical, then the most likely cause for the greater reduction in molecular weight (~12%) witnessed in extrusion Batch A compared to Batch B, relates to its longer residence time in the extruder. As reported in Section 2.2, the residence time for Batch A and Batch B were 5 min 58 s and 4 min 2 s respectively. The loss of molecular weight and the effect of residence time in PLLA during melt processing have been widely reported. However, it is a little surprising the sensitivity to molecular weight loss at such low shear rates and residence times.

#### 3.2.2. Fourier-Transform Infrared Spectroscopy

**Figure 6:**
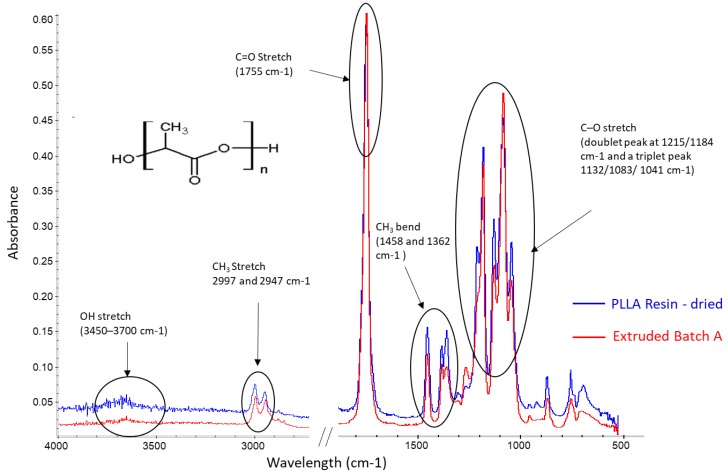
FTIR Spectra of dried resin (blue) and Batch A extruded tubing (red) in the wavelength range of 4000–500 cm^−1^.

FTIR spectra analysis of the dried resin and extruded Batch A tubing are represented in Figure 6, with their main PLLA absorption bands highlighted. The weak broad band between 3450–3700 cm^−1^, is related to the stretching of the OH (hydroxyl) group. The bands at 2997 and 2947 cm cm^−1^ correspond to CH_3_ symmetric and asymmetric stretching respectively. A strong absorption band at 1755 cm^−1^ is owed to the stretching of the C=O (carbonyl) bond. The two middle peaks at 1458 and 1362 cm^−1^ relate to the CH_3_ asymmetric and symmetric bending vibration, respectively. The doublet peak at 1215/1184 cm^−1^1 and a triplet peak 1132/1083/1041 cm^−1^ (C–O stretching vibration) indicates the nature of ester end-capped PLLA [13,31,32,33]. Whilst the spectra for extruded Batch B was not shown here, there were no discernible differences when compared to the spectra for Batch A.

A study on the effect of melt processed (injection moulded/extruded) PLLA samples, found the degradation mechanism was non-hydrolytic. This was attributed to the absence of new hydroxyl groups from carboxylic acids and alcohols (which are the products of ester hydrolysis) by using ^1^H NMR and FTIR [17]. The lack of a notable increase in the OH band in Figure 6, would suggest there has been no significant hydrolytic degradation during the extrusion process and that the degradation mechanism was most likely due to thermal degradation.

**Figure 7:**
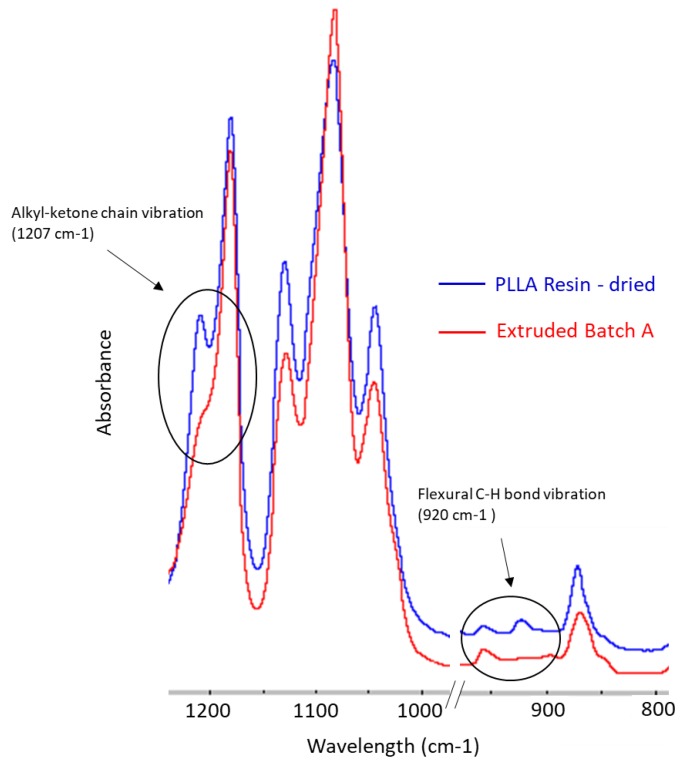
FTIR Spectra of dried resin (blue) and Batch A extruded tubing (red) in the wavelength range of 1250–800 cm^−1^.

FTIR has also been used to study the crystallisation behaviour of PLLA. Figure 7 provides a view of the absorption bands between the 1250–800 cm^−1^ wavelengths. It has been reported that the absorption bands at 1207 cm^−1^ (due to alkyl-ketone chain vibration) and 920 cm^−1^ (due to flexural C-H bond vibration) are representative of the crystalline structure of PLA [13]. When comparing the spectra of the extruded tubing to that of the PLLA resin, there is a decrease in the peak intensity at 1207 cm^−1^ and noticeable disappearance of the peak at 921 cm^−1^, which indicates a reduction in crystallinity. The peak at 955 cm^−1^ relates to the materials amorphous phase [34], however, the difference in peak intensity is negligible. These results indicate the extruded tubing has a lower degree of crystallinity than the dried resin. This is owed to the quench cooling associated with the extrusion process and will be discussed further in the thermal analysis section.

#### 3.2.3. Gas-Chromatography-Flame Ionization Detector

Gas-chromatography-flame ionization detector (GC-FID) analysis was carried out on both batches of extruded tubing to determine the effect that different processing conditions had on the percentage of residual lactide monomer present in the material. As a baseline, a sample from the dried PLLA resin pellets was also analysed. Table 3 presents the lactide weight percentage of all three samples. The dried resin has a 0.016 wt.%, compared to Batch A and B extruded tubing which was 0.366 and 0.330 wt.% respectively. There was a notable increase (~22 fold) in residual monomer created during extrusion melt processing. There was also a 10% increase in lactide monomer detected in Batch A when compared to Batch B.

The residual monomer found in polymers can be a result of the polymerization process or thermally generated from processing techniques such as melt extrusion [35]. Thermal degradation can result in random scission of the main chain or scission of the chain ends. It is postulated that chain ends are more susceptible to cleavage, which produce most of the monomers and oligomers leading to a loss in mass. However, it is a random scission that dominates the loss of molecular weight [36]. Impurities such as catalyst residues are also known to lead to depolymerisation of PLLA during melt processing [37]. It is also worth noting that despite the molecular weight loss during resin drying, the residual monomer content remained low when compared to the extruded tubing. The presence of residual monomer can serve as a plasticizer and lower the mechanical strength and thermal stability of polylactic acid [38]. The residual monomer can also have a significant effect on the in-vivo strength retention of PLA and it’s D- and L-lactide stereocomplexes, with a considerable reduction in shear strength over time, as a result of increasing levels of residual monomer [15]. Thus further highlighting the importance of melt processing on the mechanical performance of PLLA tubing, particularly in load-bearing applications. Careful selection of processing conditions must be applied to limit the increase in residual monomer during extrusion processing.

### 3.3. Thermal and Morphological Results

#### Differential Scanning Calorimetry (DSC)

**Figure 8:**
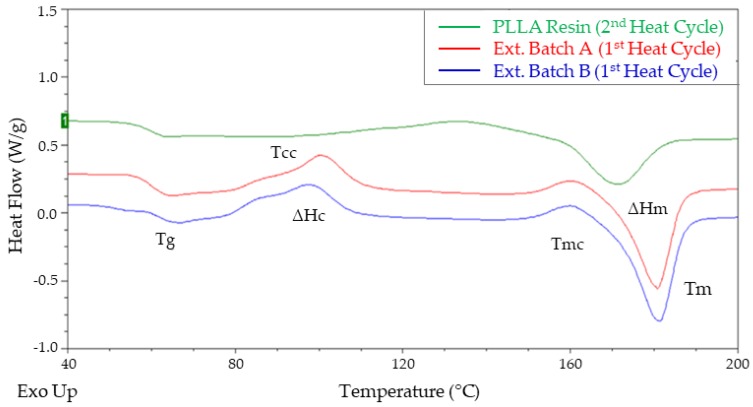
DSC curve overlay of PLLA resin and extruded Batch A & B plots. Abbreviations: T_g_: glass transition temperature; T_cc_: cold crystallisation; ΔH_c_: enthalpy of cold crystallisation; T_mc_: melt recrystallisation; T_m_: melt temperature; ΔH_m_: enthalpy of melting; X_c_: degree of crystallinity.

Thermal analysis using differential scanning calorimetry (DSC) was carried out on both batches of extruded tubing to determine the effect different processing conditions had on the thermal and morphological properties of the material. For comparison, the dried PLLA resin was also analysed. The 1st heating cycle from the DSC analysis was used to identify any differences in thermal history between extruded Batches A and B. The thermal history of the resin is removed during the extrusion processing and therefore the 2nd heating cycle was used to look at the intrinsic thermal properties of the dried resin. Figure 8 plots an overlay of the DSC curve for all 3 samples.

From the DSC curve for the PLLA resin, the glass transition temperature (T_g_) is characterized by a jump in the heat capacity (ΔC_p_) at T_g_, evident in Figure 8 as a baseline change in the heat flow. The ΔC_p_ is dependent on the crystallinity and rigid amorphous fractions of a semi-crystalline polymer [39]. T_g_ is a second order transition, where the material starts to transition from a glassy, rigid material to a soft, rubbery material [40], due to unfreezing and molecular motion of the amorphous chains. The large endothermic peak is the melting temperature (T_m_). The melting peak is a first order transition, characteristic of a semi-crystalline material and is related to the melting of the crystalline structure. The area under this peak is the enthalpy of heating (ΔH_m_) and from which the degree of crystallinity (X_c_) is calculated. These thermal properties are in-line with those reported previously for PLLA [5]. However, when the DSC curve for the PLLA resin is compared to the curves of both extruded batches, there are some notable differences.

Firstly, there is the presence of a broad double exothermic peak after T_g_, as well as smaller exothermic peak just before T_m_. The double exothermic peak represents cold crystallization (T_cc_), which is often found in polymers with slow crystallization kinetics. During rapid cooling from the molten state, the polymer does not have enough time to crystallize. However, upon re-heating above T_g_, the thermal energy provides enough molecular mobility to allow the polymer to crystallize [41]. The area under the double exothermic peak represents the enthalpy of cold crystallisation ΔH_c_. The T_cc_ double exothermic peak has been attributed to the co-existence of two kinds of amorphous regions; interlamellar amorphous regions with some order and complete amorphous regions between spherulites [42]. Alternatively, it has been proposed that the double peak is the sum of conventional cold crystallization and melt-recrystallisation of the unstable crystals formed during initial cold crystallization [43]. The small exothermic peak detected before T_m_ relates to melt recrystallisation (T_mc_). It may be caused by the presence of more than one crystallographic form; the presence of melting, re-crystallization, and re-melting; or to changes in morphology, such as lamellar thickening and crystal perfecting [44]. It has also been postulated that its presence could be due to the transition of an α’ to an α form of crystals [18].

The influence of melting conditions on thermal properties is dependent on prior melting conditions (temperature and time), heating and cooling rates and initial molecular weight. It’s important to highlight that the formation of double cold crystallisation and melt re-crystallisation peaks are significantly affected by the different heating and cooling rates applied during DSC analysis [44]. Table 4 provides a summary of the key thermal properties and calculated degree of crystallinity for the resin and both extrusion batches. From the results, there is no difference in the glass transition temperature of all three samples. However, there was an increase of ~10 °C in melt temperature with a corresponding 64% reduction in the degree of crystallinity of the extrusion batches when compared to the dried resin. Aside from a small temperature difference noted in the T_cc_ peaks, the thermal and morphological properties of both extruded batches were similar, indicating that the differences in molecular weight reported in Table 2 did not have a significant effect.

For medical device applications such as the use of implantable devices, the first stage of hydrolytic degradation is through hydrolysis of the polymers amorphous regions, resulting in molecular weight loss [4]. Therefore, the morphology of the extruded tubing will play a key role in the degradation profile of the finished device. Given the low degree of crystallinity imparted during conventional melt processing techniques such as extrusion or injection moulding, secondary processing techniques such as annealing [14,45,46,47] or biaxial expansion [48,49,50,51] are often required to increase the materials crystallinity and improve mechanical properties.

### 3.4. Mechanical Results

#### 3.4.1. Tensile Testing

**Figure 9:**
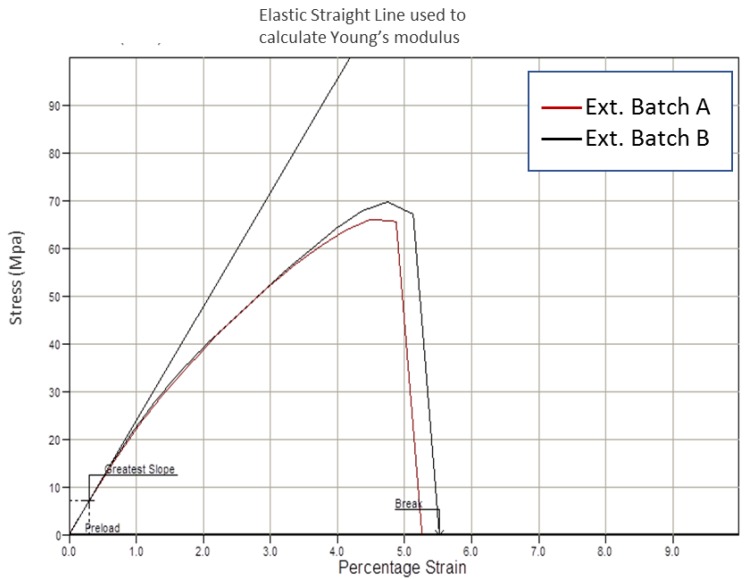
Representative tensile stress/strain curve from Batch A and B extruded tubing.

Tensile testing was performed on tubing from both extrusion batches to assess the impact melt processing had on their mechanical properties. Figure 9 provides a tensile stress/strain plot with a representative sample from both batches. The tensile curve for both batches is characteristic of a brittle material, where there is no yield point or plastic deformation. Instead, the part fails during elastic deformation, whereby the ultimate tensile strength (maximum load) and breaking strength (load at break) are effectively the same.

Table 5 provides a summary of the average tensile values for Young’s modulus, maximum tensile stress, strain at maximum load and break. Extruded Batch B had a statistically significant higher Young’s modulus (P = 0.027) and maximum tensile stress (P = 0.000) than Batch A. In practical terms, whilst a <10% difference in tensile strength and modulus may appear relatively minor, this could still have a significant effect on downstream processes such as biaxial expansion, bonding, crimping etc., as well as load-bearing applications. There was no statistical difference in the strain at maximum load (P = 0.851) for both batches. There was notable variation in the strain at break results, particularly for Batch A, however when comparing the means and standard deviations, there is no practical significance in the difference. The tensile properties presented in Table 5 are in line with those reported previously [14,18,49,52], however, a direct comparison cannot be made due to differences in process conditions, part geometry and test methods.

The tensile properties of polymers can be altered through molecular alignment. This is achieved through the orientation of the polymer molecules in the machine direction, resulting in tubes that display anisotropic behaviour. The orientation of polymer molecules results from the stress applied to the melt during processing, where the polymer melt is subjected to both shear and elongational stresses. The molecular chains are distorted from their randomly coiled state and are orientated as they pass through the die. As the extrudate exits the die, the unconstrained polymer molecules will look to revert to their natural disordered state. However, rapid cooling of the extrudate as it exits the die results in frozen-in molecular orientation and stresses. The level of drawdown, cooling and relaxation, all affect the amount of orientation in the tubing and its final properties. The extrudate will also be subjected to differential cooling rates upon exiting the die and entering the water bath. This leads to localised stresses in the tube, which can also impact its final mechanical properties.

There are numerous material characteristics that impact the mechanical properties of polymers, such as, chemical structure, molecular weight, crystallinity, and molecular orientation. As shown through DSC analysis, there were only minimal differences in crystallinity between both extruded batches. It is also unlikely that there would be a significant variation in the chemical structure or amount of orientation in both extrusion batches given the tight controls employed during the extrusion process. Therefore, the most likely contributor to the slight differences in tensile strength and stiffness is due to the higher molecular weight of Batch B over Batch A. The mechanical strength of a polymer improves with increasing molecular weight and their relationship is well understood [53]. The presence of longer polymer chains in higher molecular weight polymers, lead to an increase in chain entanglement and intermolecular forces [54]. An increase in the tensile properties of PLLA with increasing molecular weight has been reported [2]. Numerous degradation studies have shown a severe drop off in mechanical properties of PLLA as molecular weight reduces during hydrolytic degradation [55]. It is also worth noting the difference in molecular weight had no significant impact on the elongational properties of the tubing. This in part could be attributed to the molecular orientation having a stronger influence on elongation, than the observed differences in molecular weight.

#### 3.4.2. Dynamic Mechanical Analysis (DMA)

**Figure 10:**
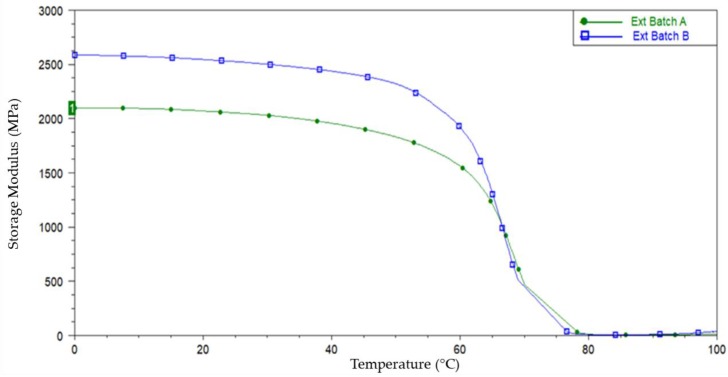
DMA plot of storage moduli from Batch A and B extruded tubes.

DMA testing was performed on a sample of tubing from both extrusion batches to assess the impact processing had on their mechanical properties. The analysis compared the storage modulus (E’) as a function of temperature between both batches. Storage modulus is a measure of the stored energy in a sample and represents the elastic portion of a viscoelastic material. Figure 10 is a DMA overlay that plots storage modulus (E’) as a function of temperature for extrusion Batches A and B. The E’ for both batches decreases slowly until a sharp drop off can be seen between 50 and 80°C. This steep drop off in E’ can be attributed to the material transitioning from a rigid glassy polymer to a rubbery polymer as it passes through its glass transition temperature. The intensity of the drop indicates that a very large fraction of the sample has been frozen in the glassy amorphous state after extrusion [34]. The DMA plot also highlights the fact that extrusion Batch B has a 22.9% higher E’ than Batch A at room temperature (21 °C), indicating an increase in stiffness. This correlates with the tensile results in Table 5, which show Batch B having a higher Young’s (elastic) modulus and maximum tensile stress than Batch A. Whilst there is a significant drop in storage modulus between 50–80°C, due the glass transition range, there is also a very subtle drop in modulus between 21 °C (room temperature), 37°C (body temperature) and 50 °C, as shown in Table 6. There is a ~4% drop between room temperature and body temperature and subsequent ~7% drop between body temperature and 50°C. This temperature dependence of mechanical properties below the glass transition temperate must be considered for any load bearing applications.

## 4. Conclusions

The tube-extrusion of high molecular weight PLLA using a microbore single screw extruder, resulted in a reduction in molecular weight as determined by GPC. The M_n_ values reduced by a range of 17–23% during processing, with resin drying contributing to approximately half of the molecular weight loss. Given the low level of moisture present in the resin (<100 ppm) post-drying, the reduction in molecular weight observed during the extrusion process can most likely be attributed to thermal degradation. This was supported by results from FTIR spectra analysis, which showed no evidence of an increase in hydroxyl groups, which are typically associated with hydrolytic degradation. It was also observed that Batch A, which was processed on the 1” extruder and had the longer melt residence time, resulted in a 12% greater molecular weight loss compared to Batch B. The loss of molecular weight and the effect of residence time in PLLA during melt processing have been widely reported. However, given the similarity in processing conditions between both batches, it was a little surprising the sensitivity to molecular weight loss at such low shear rates and melt residence times. Another important finding in this study, was the notable increase (>22 fold) in residual monomer during melt processing, as determined by GC-FID. Given the significant effect residual monomer can have on the degradation properties of PLLA, it’s clear that this must be closely monitored during melt processing.

Thermal and morphological properties of the tubing were analysed using DSC, which highlighted a reduction in crystallinity from the virgin resin (~37%) to the extruded tubes (~14%). This reduction was due to the quench cooling associated with the tube-extrusion process. In addition to these findings, the presence of cold crystallisation and melt-recrystallisation was detected in both batches of extruded tubing, which was attributed to the slow crystallisation kinetics of PLLA. This low level of crystallinity in the extruded tubing, means secondary processing such as annealing, or strain induced crystallisation are most likely required. The differences in molecular weight from Batch A and B did not impact the thermal or morphological properties of the tubing.

The mechanical properties of both batches were examined using uniaxial tensile testing and dynamic mechanical analysis. As reported by many other authors, both analytical techniques highlighted the material to be brittle with moderate stiffness and strength when compared to other biodegradable polymers. Tensile testing reported Young’s modulus values in the range of 2400–2500 MPa, the maximum tensile stress in the range of 66–70 MPa and strain at break in the region of <5.5%. The lack of any strain hardening or softening on the extruded tubing is an important consideration for downstream processing such as biaxial expansion.

Batch B exhibited slightly higher stiffness and tensile strength than Batch A, which can be attributed to its higher molecular weight. Interestingly the difference in molecular weight had no notable impact on elongational properties. This in part could be owed to the molecular orientation having a stronger influence on elongation, than slight differences in molecular weight. The temperature dependence of mechanical properties below T_g_ was also confirmed through the use of DMA.

From the findings in this study, the role that low shear microbore extrusion plays in determining the thermal, chemical, morphological and mechanical properties of PLLA tubing is better understood. Whilst many of the results are in agreement with the findings of other researchers, there were some key learnings. Careful consideration of process parameters and tooling configuration must be considered, given the susceptibility of PLLA to thermal degradation during melt processing, at low shear rates. Even at these low shear rates, the residence time in the extruder plays a crucial role in determining the final molecular weight of the material and must be controlled, whilst the level of residual monomer in the finished tubing must be carefully monitored. Small differences in molecular weight (12%) were shown to impact tensile strength and modulus, with no notable effect on elongation, further highlighting the importance of controlling molecular orientation during the extrusion process. There was also an observed temperature-dependence of mechanical properties below the glass transition temperature.

## Figures and Tables

**Table 1:** Extrusion screw geometry details for both the 0.75” and 1” screws.

Screw Dia.	Feed Depth	Metering Depth	Compression Ratio	l/d Ratio	Feed Section	Compression Section	Metering Section
0.75”	0.150	0.050”	3:1	24/1	8 Flights	8 Flights	8 Flights
1“	0.144	0.046”	3.1:1	24/1	8 Flights	8 Flights	8 Flights

**Table 2:** GPC results of the PLLA resin (as received and dried) and extrusion Batches A and B.

Sample Reference	M_n_ (g/mol)	M_w_ (g/mol)	PDI
PLLA Resin - as received	200,073	430,409	2.16
PLLA Resin - dried	177,662	419,715	2.36
Extruded Batch A	146,177	367,575	2.51
Extruded Batch B	165,472	413,439	2.50

**Table 3:** GC-FID results presenting the lactide weight percentage of the PLLA resin (dried) and extrusion Batches A and B.

Sample Reference	Lactide wt.%
PLLA Resin - dried	0.016
Extruded Batch A	0.366
Extruded Batch B	0.330

**Table 4:** Summary of thermal properties of dried PLLA dried resin and Batch A and B extruded tubing.

Batch Reference	T_g_ (°C)	ΔC_p_ J/(g°C)	T_cc_ (°C)	ΔH_c_ (J/g)	T_mc_ (°C)	T_m_ (°C)	ΔH_m_ (J/g)	X_c_ (%)
Resin – Dried	60.7	0.49	-	-	-	171.0	34.5	37.1
Extruded Batch A	61.4	0.95	100.9	27.6	159.9	180.6	39.8	13.2
Extruded Batch B	61.6	0.62	97.7	27.4	159.4	181.5	40.2	13.8

**Table 5:** Summary of tensile properties of Batch A and B extruded tubing.

Batch Reference		Young’s Modulus (MPa)	Maximum Tensile Stress (MPa)	Strain at Maximum Load (%)	Strain at Break (%)
Extrusion Batch A	X (σ)	2408 (66.6)	66.6 (1.37)	4.47 (0.288)	5.54 (1.17)
Extrusion Batch B	X (σ)	2521 (80.8)	70.5 (0.94)	4.44 (0.281)	5.38 (0.33)

**Table 6:** DMA Storage Moduli at different temperatures for batch A and B extruded tubing.

Batch Reference	Storage Modulus (MPa) @ 21 °C	Storage Modulus (MPa) @ 37 °C	Storage Modulus (MPa) @ 50 °C
Extrusion Batch A	2067	1984	1831
Extrusion Batch B	2541	2460	2321
